# Multifocal Peritoneal Mucinous Carcinoma Presenting as Small Bowel Obstruction: A Case Report

**DOI:** 10.7759/cureus.110258

**Published:** 2026-06-04

**Authors:** Haytham Mohammed Alzinati, Ahmed Zaki, Abdulmalek Ayman Arbach, Ahmed Fathi Mohamed Alsehily, Ahmed Elsayed Ibrahim Mattar, Ammar Mohamed Saleih Abdullah Basheir

**Affiliations:** 1 General Surgery, Saudi German Hospital, Hail, Hail, SAU; 2 General Surgery, Ibn Sina University, Khartoum, SDN

**Keywords:** gastrointesinal malignancy, laparoscopy, mucinous carcinoma, peritoneal carcinomatosis, small bowel obstruction

## Abstract

Mucinous carcinoma involving the small bowel is a rare cause of intestinal obstruction and a diagnostically challenging entity. When multifocal mucinous deposits involve the small bowel and abdominal wall, establishing the diagnosis and identifying the site of origin requires an integrated clinicopathological approach. We present a case of a 42-year-old woman who presented with acute small bowel obstruction. Laparotomy revealed multiple lesions involving the ileum and cecal region. The primary obstructing lesion was located in the mid-ileum with proximal bowel dilatation and distal collapse. Additional non-obstructing lesions were identified approximately one meter distal to the first lesion and near the ileocecal valve, along with a cecal lesion adherent to the right fallopian tube. Histopathological examination demonstrated multifocal mucinous carcinoma with abundant extracellular mucin pools containing malignant epithelial cells infiltrating the intestinal wall and fibroadipose tissue. This case highlights the diagnostic challenge posed by multifocal mucinous carcinoma presenting as bowel obstruction. Awareness of this rare presentation is important for surgical decision-making and postoperative oncologic evaluation.

## Introduction

Intestinal obstruction is a common surgical emergency, with adhesions representing the leading etiology [1-2]. However, in a subset of patients, an underlying neoplastic process must be considered and actively sought. Malignant conditions, arising from primary or metastatic cancers, account for approximately 5% to 20% of cases, with the most common causes being adenocarcinomas, carcinoid tumors, and gastrointestinal stromal tumors [1]. Among the less common causes of small bowel obstruction, peritoneal surface malignancies warrant particular attention, as they may produce bowel obstruction through extrinsic compression, adhesive tumor deposits, or direct intraluminal invasion [3].

Small bowel tumors themselves are rare, representing less than 5% of all gastrointestinal malignancies [4-5]. Among these, mucinous carcinomas constitute an uncommon histological subtype and are more frequently encountered in the appendix and colorectum than in the small intestine. Primary or multifocal mucinous involvement of the small bowel is therefore exceptionally rare [6-7].

Peritoneal surface malignancies constitute a heterogeneous group of neoplasms that arise primarily from or spread secondarily to the peritoneum, including peritoneal carcinomatosis from gastrointestinal and gynecologic primaries, pseudomyxoma peritonei, and rare primary peritoneal mucinous adenocarcinomas [3,8]. Bowel obstruction was the presenting symptom in 19% of cases in a large retrospective series of patients with non-gynecologic peritoneal carcinomatosis [8]. Moreover, the insidious nature of peritoneal mucinous disease often results in delayed diagnosis, as symptoms may remain vague and nonspecific until a considerable tumor burden has accumulated [8].

We report the case of a 42-year-old woman presenting with small bowel obstruction who was ultimately diagnosed with multifocal mucinous carcinoma involving the abdominal wall, ileum, and ileocecal part of the bowel. This case highlights the diagnostic challenges associated with peritoneal mucinous carcinomatosis and emphasizes the importance of surgical exploration and comprehensive histopathological analysis.

## Case presentation

A 42-year-old woman presented to the emergency department with a history of acute colicky abdominal pain, vomiting, and constipation of five-day duration. She had been admitted four months earlier with similar symptoms. At that time, abdominal imaging demonstrated features of small bowel obstruction without an identifiable mass lesion. She was managed conservatively with bowel rest, nasogastric decompression, and supportive treatment, resulting in immediate recovery and discharge. The patient had no significant past medical history, apart from having undergone three cesarean sections.

**Figure 1:**
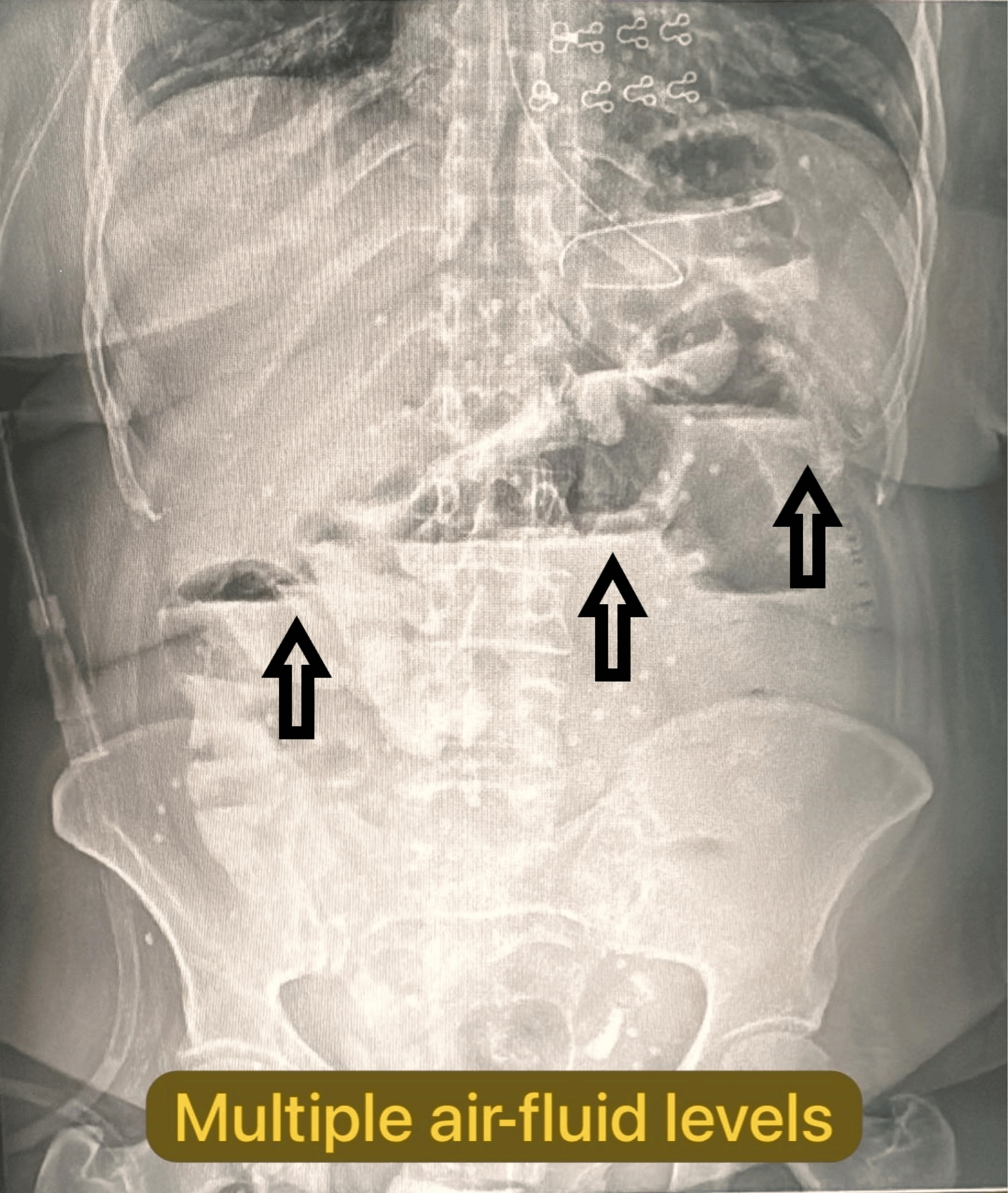
A plain abdominal radiograph demonstrating multiple dilated small bowel loops with air-fluid levels, consistent with proximal small bowel obstruction

During the current admission, the abdomen was diffusely distended and tender, with clinical features consistent with bowel obstruction. Vital signs were stable, with temperature 37.1°C, pulse 78, BP 110/70, and O₂ saturation 98%. Laboratory investigations on admission revealed a mild microcytic hypochromic anemia (hemoglobin 10.0 g/dL, hematocrit 31.5%, mean corpuscular volume, or MCV, 75.2 fL, and mean corpuscular hemoglobin, or MCH, 24.0 pg), a mildly alkalotic blood gas profile (venous pH 7.44 and HCO₃⁻ 26.5 mmol/L), and elevated lactate levels (10.5 mmol/L). A plain abdominal radiograph demonstrated multiple dilated small bowel loops with air-fluid levels, consistent with proximal small bowel obstruction (Figure 1).

**Figure 2:**
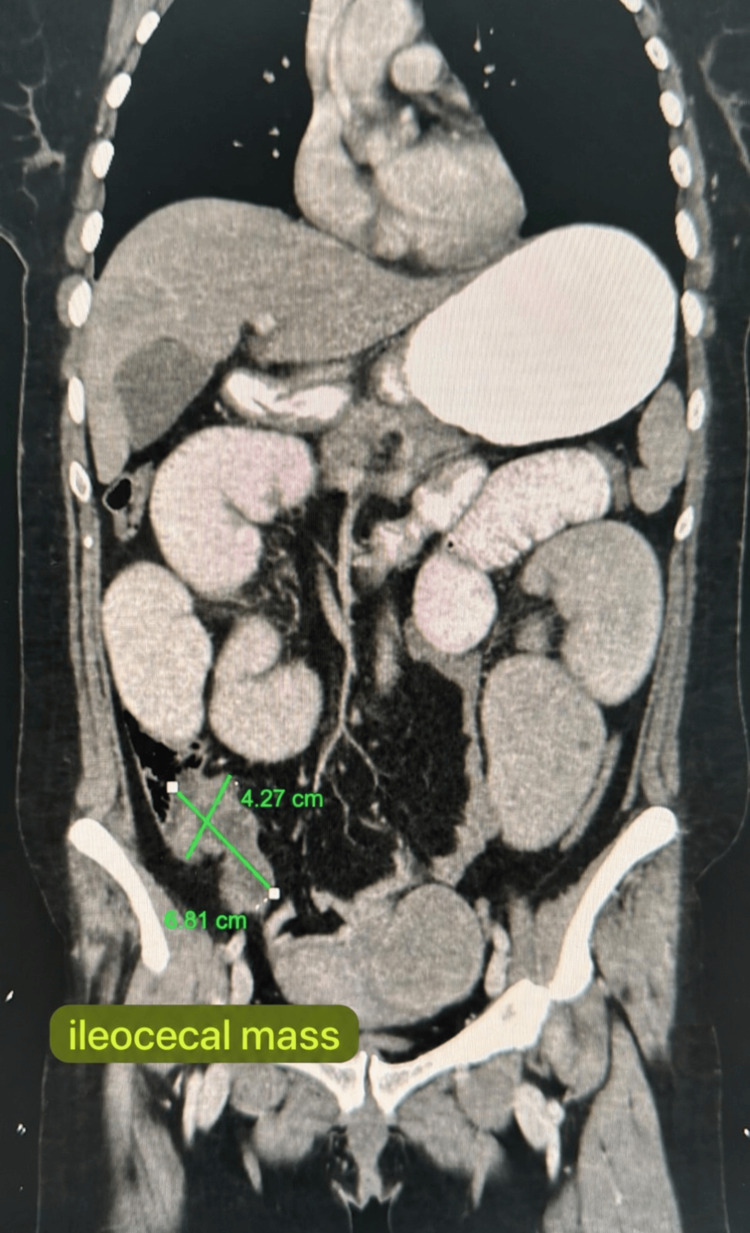
A CT scan of the abdomen demonstrating an ileocecal mass

Computed tomography (CT) of the abdomen and pelvis with contrast demonstrated dilated ileum and a heterogeneous soft-tissue mass in the right lower quadrant centered at the ileocecal region (Figure 2). The solid abdominal organs (liver, spleen, kidneys, pancreas, gallbladder, and adrenal glands) were radiologically unremarkable, and no lymphadenopathy was detected.

**Figure 3:**
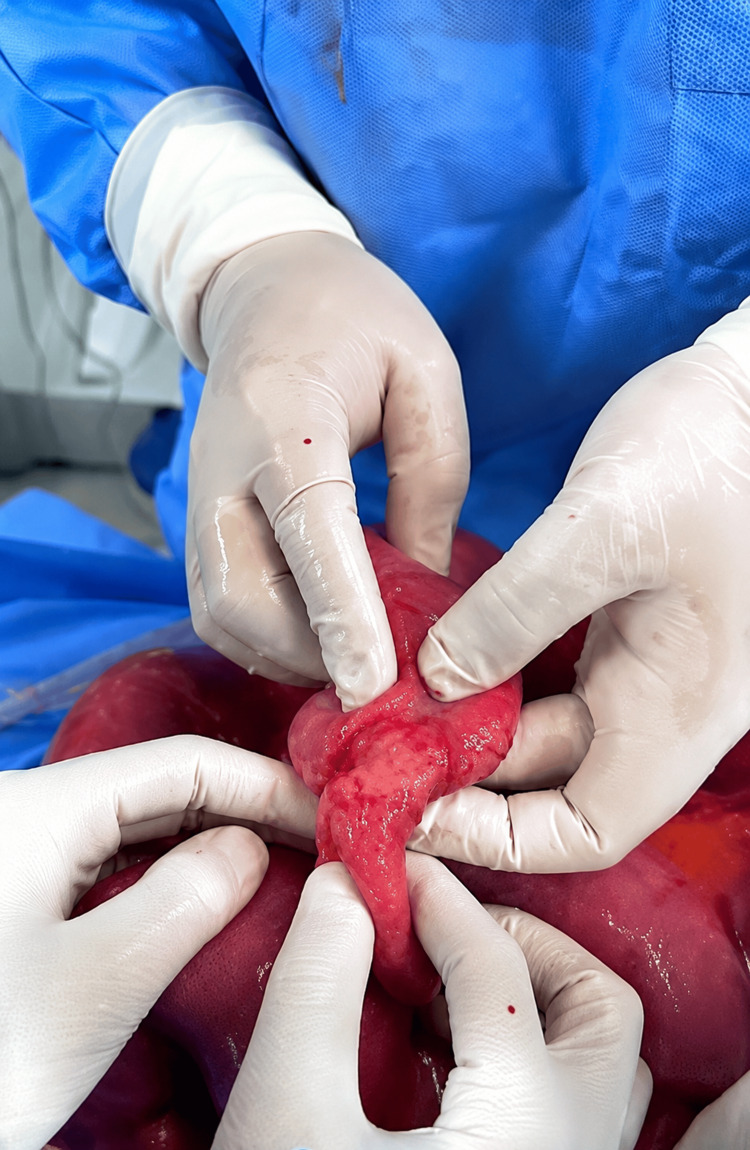
An intraoperative photograph showing the obstructing mid-ileal mass

After initial conservative management with nasogastric decompression, intravenous fluid resuscitation, and bowel rest, a decision was made to proceed with operative intervention. Laparotomy was performed, commencing at the duodenojejunal junction and proceeding distally. The first lesion was identified in the mid-ileum, approximately two meters proximal to the ileocecal valve. This was a mass with necrotic-appearing tissue and unhealthy surrounding mesentery. A clear transition zone was identified at this level, with markedly dilated proximal bowel loops and completely collapsed distal loops, confirming this as the definitive source of obstruction (Figure 3).

Continued systematic exploration revealed two further lesions. A second ileal mass was identified approximately one meter distal to the first, located 30 cm proximal to the ileocecal valve; this lesion did not obstruct the bowel lumen. A third lesion was identified as a cecal mass occupying the right iliac fossa, with the right fallopian tube found adherent. On further exploration, the appendix and other segments were grossly normal, with no evidence of primary pathology or metastatic deposits.

Given the diagnostic uncertainty at the time of surgery, a decision was made to perform a resection of the obstructing lesion and to exteriorize the bowel as a double-barrel ileostomy to decompress the cecum, along with relieving the obstruction. The excised specimens from the lesions were sent for analysis to establish a tissue diagnosis.

Histopathological examination of the obtained specimens revealed similar morphology across all lesions. Microscopically, all specimens demonstrated a tumor composed of abundant extracellular mucin pools containing floating malignant epithelial cells with an infiltrative growth pattern involving the intestinal wall and fibroadipose tissue. The final histopathological diagnosis was mucinous carcinoma involving the abdominal wall and small intestine (multifocal deposits).

## Discussion

Small bowel tumor is a rare malignancy, accounting for less than 5% of all gastrointestinal tumors, and its mucinous variant is even less frequently encountered [9]. Mucinous neoplasms are rare and predominantly originate from the appendix; however, there are very few cases of extra-appendiceal origin [10]. Multifocal mucinous involvement of the small bowel and abdominal wall, as seen in the present case, adds a further layer of diagnostic complexity, as it raises the question of whether multiple separate primary tumors or a single primary tumor with peritoneal seeding is responsible.

Multifocal mucinous adenocarcinoma involving the small intestine is an extremely rare malignancy. Mucinous carcinomas are a distinct histological subtype defined by the presence of abundant extracellular mucin comprising more than 50% of the tumor volume [11]. They may originate from a variety of sites, including the colorectum, appendix, ovary, stomach, pancreas, and, less commonly, the small intestine. In advanced-stage disease, tumor adherence to small bowel surfaces is well-recognized and can precipitate obstruction.

In this case, initial laboratory investigations on admission revealed a microcytic hypochromic anemia with reduced hemoglobin, MCV, and MCH, along with an elevated red cell distribution width (RDW) of 15.0%, consistent with iron-deficiency anemia likely reflecting chronic occult blood loss in the context of an underlying malignant process. Additionally, venous blood gas analysis demonstrated a mildly alkalotic pH with a normal PCO₂ and a marginally elevated bicarbonate level of 26.5 mmol/L, consistent with a compensated metabolic alkalosis secondary to prolonged vomiting and gastric acid loss. Notably, the lactate level was significantly elevated at 10.5 mmol/L, indicating clinically significant tissue hypoperfusion attributable to the bowel obstruction and reinforcing the urgency of operative intervention.

The obstructive mechanism may be extrinsic compression from tumor deposits, adhesion formation secondary to peritoneal inflammation, or direct invasion of the bowel wall in advanced disease. In the present case, the mass at the proximal jejunum (20 cm from the duodenojejunal junction) directly obstructed the bowel lumen, while the other deposits represented satellite peritoneal disease.

This case illustrates several important diagnostic and clinical challenges associated with peritoneal mucinous carcinomatosis presenting as small bowel obstruction. The patient had a prior admission with similar obstructive symptoms without identifiable radiological findings. At that time, the patient had responded temporarily to conservative management, suggesting an intermittent or evolving obstructive process before definitive diagnosis. The radiological non-specificity and imaging limitations in peritoneal mucinous disease have been reported [3,8]. Peritoneal implants smaller than 5-10 mm are frequently missed, particularly on the small bowel serosa. Mucin within the peritoneal cavity usually presents as low-attenuation fluid with some soft tissue attenuation. Therefore, the low-attenuation nature of mucinous deposits on non-contrast CT makes them closely approximate fluid density, making their differentiation from ascites and adjacent bowel loops challenging [12]. The absence of an obvious primary tumor on preoperative CT is a recognized feature of peritoneal mucinous carcinomatosis, particularly when the disease burden is predominantly peritoneal with small or occult primaries [13-15]. The initial contrast-enhanced CT in this patient identified the dilated bowel and a transition point but did not detect a discrete primary mass, a finding consistent with the known limitations of CT in characterizing early or small mucinous neoplasms [13-15].

While intraoperative findings raised suspicion for carcinomatosis, the diagnosis is limited by the lack of comprehensive immunohistochemical profiling and long-term follow-up, restricting definitive determination of tumor origin and long-term prognosis. The multifocal distribution across discrete anatomical sites favored secondary metastatic or peritoneal spread of mucinous carcinoma and recommended immunohistochemical staining to determine the primary site of origin [16-17]. Immunohistochemistry plays a role in establishing the likely primary origin of metastatic mucinous carcinomas. Established markers include cytokeratin 7 (CK7), CK20, CDX2, SATB2, PAX8, and hormone receptors (estrogen receptor and progesterone receptor), each contributing to a diagnostic profile that may point towards a colorectal, appendiceal, ovarian, or other origin [16-17].

## Conclusions

This case highlights the diagnostic and surgical challenges associated with multifocal mucinous carcinoma presenting as small bowel obstruction. While the intraoperative and histopathological findings are highly suggestive of peritoneal dissemination of a mucinous malignancy, the diagnosis remains limited in the absence of comprehensive immunohistochemical analysis.

Early multidisciplinary involvement, including surgical, pathological, and oncological input, remains essential to guide further evaluation and management. Given the rarity of multifocal mucinous involvement of the small bowel, this case contributes to the limited available literature while emphasizing the need for cautious interpretation of diagnostic findings.
